# HBV subgenotypes F1b and F4 replication induces an incomplete autophagic process in hepatocytes: Role of BCP and preCore mutations

**DOI:** 10.1371/journal.pone.0197109

**Published:** 2018-05-08

**Authors:** María Mercedes Elizalde, Paula Soledad Pérez, Ina Sevic, Daniel Grasso, Alejandro Ropolo, Luciana Barbini, Rodolfo Héctor Campos, María Inés Vaccaro, Diego Martín Flichman

**Affiliations:** 1 Cátedra de Virología, Departamento de Microbiología, Inmunología, Biotecnología y Genética, Facultad de Farmacia y Bioquímica, Universidad de Buenos Aires, Buenos Aires, Argentina; 2 Consejo Nacional de Investigaciones Científicas y Técnicas (CONICET), Buenos Aires, Argentina; 3 Instituto de Bioquímica y Medicina Molecular, Departamento de Fisiopatología, Facultad de Farmacia y Bioquímica, Universidad de Buenos Aires, Buenos Aires, Argentina; 4 Departamento de Química, Facultad de Ciencias Exactas y Naturales, Universidad Nacional de Mar del Plata, Mar del Plata, Argentina; Toho Daigaku, JAPAN

## Abstract

Hepatitis B virus (HBV) genotypes and mutants have been associated with differences in clinical and virological characteristics. Autophagy is a cellular process that degrades long-lived proteins and damaged organelles. Viruses have evolved mechanisms to alter this process to survive in host cells. In this work, we studied the modulation of autophagy by the replication of HBV subgenotypes F1b and F4, and the naturally occurring mutants BCP and preCore. HBV subgenotypes F1b and F4 replication induced accumulation of autophagosomes in hepatoma cells. However, no autophagic protein degradation was observed, indicating a blockage of autophagic flux at later stages. This inhibition of autophagy flux might be due to an impairment of lysosomal acidification in hepatoma cells. Moreover, HBV-mediated autophagy modulation was independent of the viral subgenotypes and enhanced in viruses with BCP and preCore naturally occurring mutations. These results contribute to understand the mechanisms by which different HBV variants contribute to the pathogenesis of HBV infections. In addition, this study is the first to describe the role that two highly prevalent naturally occurring mutations exert on the modulation of HBV-induced autophagy.

## Introduction

Hepatitis B virus (HBV) is an important human pathogen that chronically infects approximately 257 million people worldwide [1]. HBV chronic infection is associated with the development of severe liver diseases, including liver cirrhosis and hepatocellular carcinoma (HCC), which causes approximately 887.000 deaths annually.

The evolution of HBV infection relies on a finely balanced and complex interaction between the virus and the host immune system. Epidemiological data have increasingly associated HBV genotypes and mutants with differences in clinical and virological characteristics, such as severity of liver disease and response to antiviral therapies [2–5].

HBV has been classified in ten genotypes (A-J) and multiple subgenotypes (sgts) [6]. The genotypes and sgts have a different ethnic-geographical distribution [7]. In Argentina, sgts F1b and F4, likely originated in Amerindian populations, are the most prevalent. Previous studies have shown differences in clinical and virological characteristics between these two sgts. Individuals infected with HBV sgt F1b seroconvert e antigen (HBeAg) later in life than those infected with sgt F4 [8]. In addition, recent studies have shown that sgt F1b is the main responsible of the new acute and chronic infections in Argentina [8–10]. Moreover, growing evidence has shown a close association of sgt F1b with more severe course of chronic HBV infections, and a high correlation between sgt F1b and the development of HCC exists among Alaska native people [11–13].

During the course of chronic infection, HBV genome accumulates changes that allow its adaptation to a selective environment. These mutations exert various effects on viral replication, clinical features and pathogenesis [3]. In particular, in the late stage of chronic infection, mutations in basal core promoter (BCP) and preCore regions occur. The predominant mutation in BCP is the double mutation at A1762T/G1764A. It has been shown that this double mutation down-regulates preCore RNA transcription and HBeAg expression, and increases viral replication rate [14]. Regarding preCore region, the most common mutation is G1896A, which abolishes HBeAg expression [15]. Both mutations have been associated with progression of HBV chronic infection [16–18], although the mechanisms underlying this process are unknown.

Autophagy is an intracellular process that degrades cytosolic proteins and damaged organelles. When autophagy is induced, an isolation membrane encloses a portion of cytoplasm, forming a double-membraned vesicle called autophagosome. The autophagosome then fuses with the lysosomes to generate an autolysosome, leading to the degradation of the enclosed materials by lysosomal enzymes [19]. Autophagy plays an important role in maintaining cellular homeostasis and cell survival under stress conditions [20]. Moreover, the dysfunction of autophagy has been implicated in multiple pathological conditions including neurodegenerative diseases, cancer, inflammatory diseases, and infectious diseases [21]. Autophagy has also been implicated in innate and adaptive immune responses against intracellular microbial pathogens [22]. Thus, many viruses have evolved mechanisms to alter this process in order to survive in the host cells.

It has been demonstrated that HBV can enhance and exploit autophagy for its DNA replication [23–26]. However, it is unknown if HBV replication of the native-American sgts F1b and F4 induces autophagy in hepatocytes, and to what extent, BCP and preCore mutants can differentially modulate this mechanism. Alteration of this intracellular process by HBV variants may play an important role in viral pathogenesis.

The aim of this study was to analyze whether HBV replication of sgts F1b and F4 modulates autophagy in human hepatoma cells, and to study the effect of BCP and preCore mutations in the regulation of this process.

## Materials and methods

### Cell culture

Human hepatoma cell line Huh-7 (JCRB Cell Bank #0403) was cultured in Dulbecco’s modified Eagle’s medium (DMEM, GIBCO, USA) supplemented with 10% fetal bovine serum (Internegocios, Argentina), 1 mM nonessential aminoacids (GIBCO, USA), 0.15% sodium bicarbonate, 100 UI/ml penicillin and 100 μg/ml streptomycin. Cells were maintained at 37°C in a humidified atmosphere containing 5% CO_2_.

### Plasmids

Vector pUC19 containing full-length HBV genomes of sgts F1b and F4 with the wt sequence, the double BCP (A1762T/G1764A; BCPdm) and the preCore (G1896A) mutations were analyzed in this study (S1 Fig). The generation of these plasmids was previously described [27].

The mRFP-GFP-LC3 plasmid was a kind gift of Dr. María Isabel Colombo (Laboratorio de Biología Celular y Molecular, Instituto de Histología y Embriología, Facultad de Ciencias Médicas, Universidad Nacional de Cuyo).

### Transient transfection

In order to simulate viral variability, a mix of 10 to 20 clones (pUC19-full-length HBV genomes) of each viral variant was used in each experiment.

As previously described, full-length linear HBV genomes were excised from the plasmid by restriction enzyme digestion with 5 U of BspQI (New England Biolabs, USA) at 50°C [27]. The 3.2-kb fragments were gel purified with PureLink Quick Gel Extraction Kit (Invitrogen, USA), according to the manufacturer’s instructions and the DNA was quantified spectrophotometrically.

For transfections, cells were seeded in 24 or 6 well plates and grown to 60–70% confluence. Transfections were carried out using X-tremeGene 9 transfection reagent (Roche, Germany), according to the manufacturer’s recommendations. Empty vector pUC19 was used as control. Cells were maintained at 37°C in 5% CO_2_ atmosphere. After 6 h incubation, medium was replaced, and cultures were incubated for 48 h. Cells were treated with 100 μM chloroquine (CQ; Sigma, USA) when indicated.

### Autophagy induction by cell starvation

Cells were washed 3 times with pre-warmed PBS and then incubated in Earle's balanced salts solution (EBSS; 117 mM NaCl, 1.8 mM CaCl_2_, 0.8 mM MgSO_4_, 5.3 mM KCl, 5.6 mM glucose, 1 mM NaH_2_PO_4_-H_2_O and 26 mM NaHCO_3_, pH 7.4) at 37°C for 2 h.

### Transmission electron microscopy (TEM)

Forty-eight hours after transfection, approximately 5 x 10^6^ cells were scraped, washed twice with PBS and fixed for TEM with 2% glutaraldehyde in phosphate buffer (80 mM Na_2_HPO_4_ and 25 mM NaH_2_PO_4_, pH 7.4) at 4°C for 2 h. The fixing solution was removed, and the cell pellet was washed with phosphate buffer every 30 minutes at 4°C. The cells were post-fixed in 1% osmium tetroxide and embedded in an epoxy Spurr resin. Thin sections (0.5 μm) were stained with toluidine blue and observed by light microscopy in order to select fields. Ultrathin sections were mounted on 200 mesh copper grids and stained with uranyl acetate and lead citrate. The grids containing the sections were observed on a Zeiss EM 109T transmission electron microscope [Laboratorio Nacional de Investigación y Servicio de Microscopía Electrónica, (LANAIS-MIE, UBA-CONICET)] at 80 kV and photographed (Gatan ES1000W). Autophagosome and autolysosome number were quantified using ImageJ software (Wayne Rasband, NIH, USA).

### Immunofluorescence

Cells were grown on coverslips and transfected with the different full-length HBV genomes. Forty-eight hours after transfection, cells were fixed with 100% methanol for 15 min at -20°C. The fixed cells were wash 3 times in cold PBS for 5 min and blocked with 1% bovine serum albumin (BSA), 0.1% Triton X-100 in PBS for 1 h. Cells were incubated with the primary antibody anti-LC3 (1:200, Cell Signaling Technology, USA) overnight at 4°C, followed by incubation with Alexa Fluor 488-conjugated anti-rabbit secondary antibody (1:200; Cell Signaling Technology, USA) for 1 h at room temperature in the dark. Cells were washed 3 times in PBS and nuclei were stained with 1 μg/ml DAPI (Sigma, USA) for 5 min. Coverslips were mounted with Vectashield (Vector Laboratories, USA), examined under a fluorescent microscope (Olympus BX50) and photographed. Image analysis was performed with ImageJ software (Wayne Rasband, NIH, USA).

### Western Blot analysis

For total protein extraction, approximately 2 x 10^6^ adherent and detached cells were harvested, washed with PBS and resuspended in lysis buffer [20 mM Tris, 1 mM EDTA, 150 mM NaCl, 1% Triton, and protease inhibitor cocktail (Sigma, USA)]. The cells were lysed by 3 freeze/thawing cycles. Lysates were centrifuged, and supernatants were harvested. Total protein concentration was determined using the Bradford protein assay. Equal amounts of proteins were loaded on 15% SDS-polyacrylamide gels and transferred to polyvinylidene difluoride (PVDF) membranes (Hybond, GE Healthcare, UK) by electroblotting at 100 V for 60 min. The membranes were blocked for 1 h in 5% non-fat milk in Tris-buffered saline (20 mM Tris and 150 mM NaCl, pH 7.6) containing 0.1% Tween-20 (TBST) for 1 h at room temperature, followed by incubation with the specific primary antibody: rabbit polyclonal anti-LC3 (1:1000, Cell Signaling Technology, USA), rabbit polyclonal p62 (1:1000, Cell Signaling Technology, USA) and mouse monoclonal anti-β-actin (0.5 μg/ml, Cell Signaling Technology, USA), overnight at 4°C. The membranes were washed 3 times with TBST and incubated with the corresponding horseradish peroxidase-conjugated secondary antibody: goat anti-mouse (1:30000, Santa Cruz Technology, USA) or goat anti-rabbit (1:10000, Santa Cruz Biotechnology, USA) for 1 h at room temperature. Protein specific bands were visualized using an enhanced chemiluminescence (ECL) system (GE Healthcare, UK) by autoradiography. The quantification was performed using ImageJ analysis software (Wayne Rasband, NIH, USA). β-actin detection was used as total protein loading control.

### Acridine orange staining (AO)

Cells were grown on coverslips and transfected with the different full-length HBV genomes. Forty-eight hours after transfection, cells were fixed with 4% formaldehyde for 10 min at room temperature and stained with 4 μg/ml AO (Sigma, USA) for 15 min at 37°C. After washing with PBS to remove excess of AO, cells were visualized by fluorescent microscopy (Olympus BX50) and photographed. Image analysis was performed with ImageJ software (Wayne Rasband, NIH, USA).

### Tandem fluorescent mRFP-GFP-LC3 assay

Cells grown in coverslips were co-transfected with full-length HBV genomes and mRFP-GFP-LC3 plasmid. Forty-eight hours after transfection, cells were fixed with 4% formaldehyde for 10 min at room temperature, washed 3 times in cold PBS and visualized by fluorescent microscopy (Olympus BX50) and photographed. Image analysis was performed with ImageJ software (Wayne Rasband, NIH, USA).

### Statistical analysis

All experiments were independently performed three times. Statistical significance was determined using a two-tailed Student *t* test or one-way analysis of variance (ANOVA) followed by *post hoc* Bonferroni test when applicable. A value of *p* < 0.05 was considered to be statistically significant. Results were expressed as mean ± standard deviation. All analyses were performed using GraphPad Prism 5.01 software (GraphPad Software, San Diego, CA, USA).

## Results

### HBV replication of sgts F1b and F4 induces accumulation of autophagosomes

**Fig 1:**
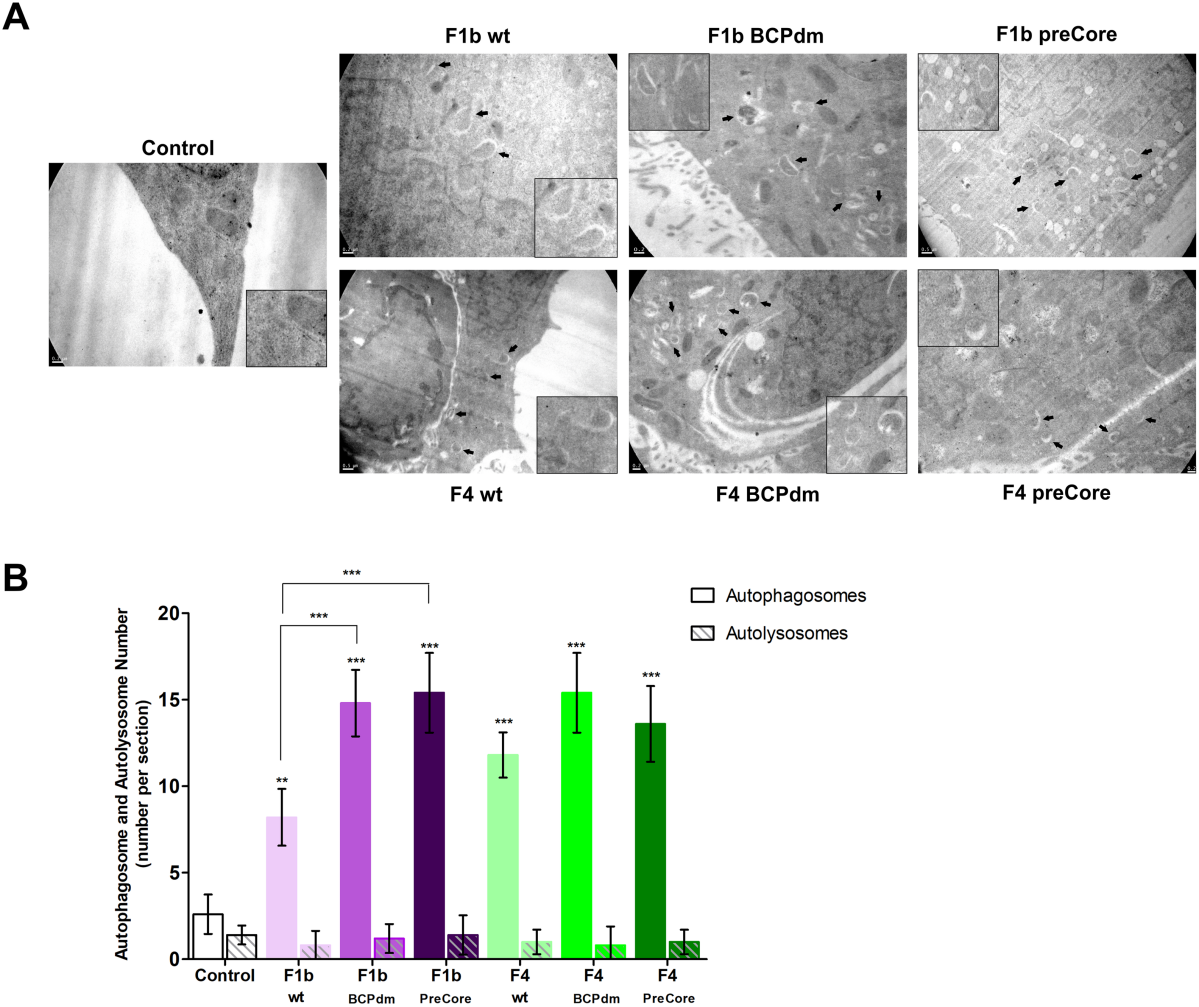
Detection of autophagic vacuoles by transmission electron microscopy in cells transfected with full-length HBV genomes. (A) Representative transmission electron micrographs of Huh-7 cells transfected with pUC19 empty vector (control), F1b wt, F1b BCPdm, F1b preCore, F4 wt, F4 BCPdm and F4 preCore variants. Arrows indicate representative autophagic vacuoles. Inset: enlargement of autophagic vacuoles in the electron micrograph. (B) Quantification of autophagic vacuoles (autophagosomes and autolysosomes). Shown values represent the mean ± standard deviation of three independent experiments. Statistically significant changes compared to control cells are indicated with asterisks above bars and statistically significant changes between the viral variants are indicated with asterisks above brackets. *** *p* < 0.0001.

To investigate whether HBV replication of sgts F1b and F4 wt sequences and BCP/preCore mutants could modulate autophagy, the presence of autophagic vacuoles was detected at the ultrastructural level by transmission electron microscopy. In comparison to control transfected cells whose autophagic vacuoles were rarely observed, an increased number of autophagosomes (double-limiting membrane compartments with cytoplasmic material and/or organelles) was detected in cells transfected with wt and mutants of sgts F1b and F4 HBV genomes. In addition, a significant increase in the number of autophagosomes was observed with sgt F1b BCPdm and preCore variants, compared to sgt F1b wt. However, the number of autolysosomes (single-limiting membrane compartments containing cytoplasmic materials at various stages of degradation) did not increase in HBV transfected cells in relation to control cells (Fig 1).

**Fig 2:**
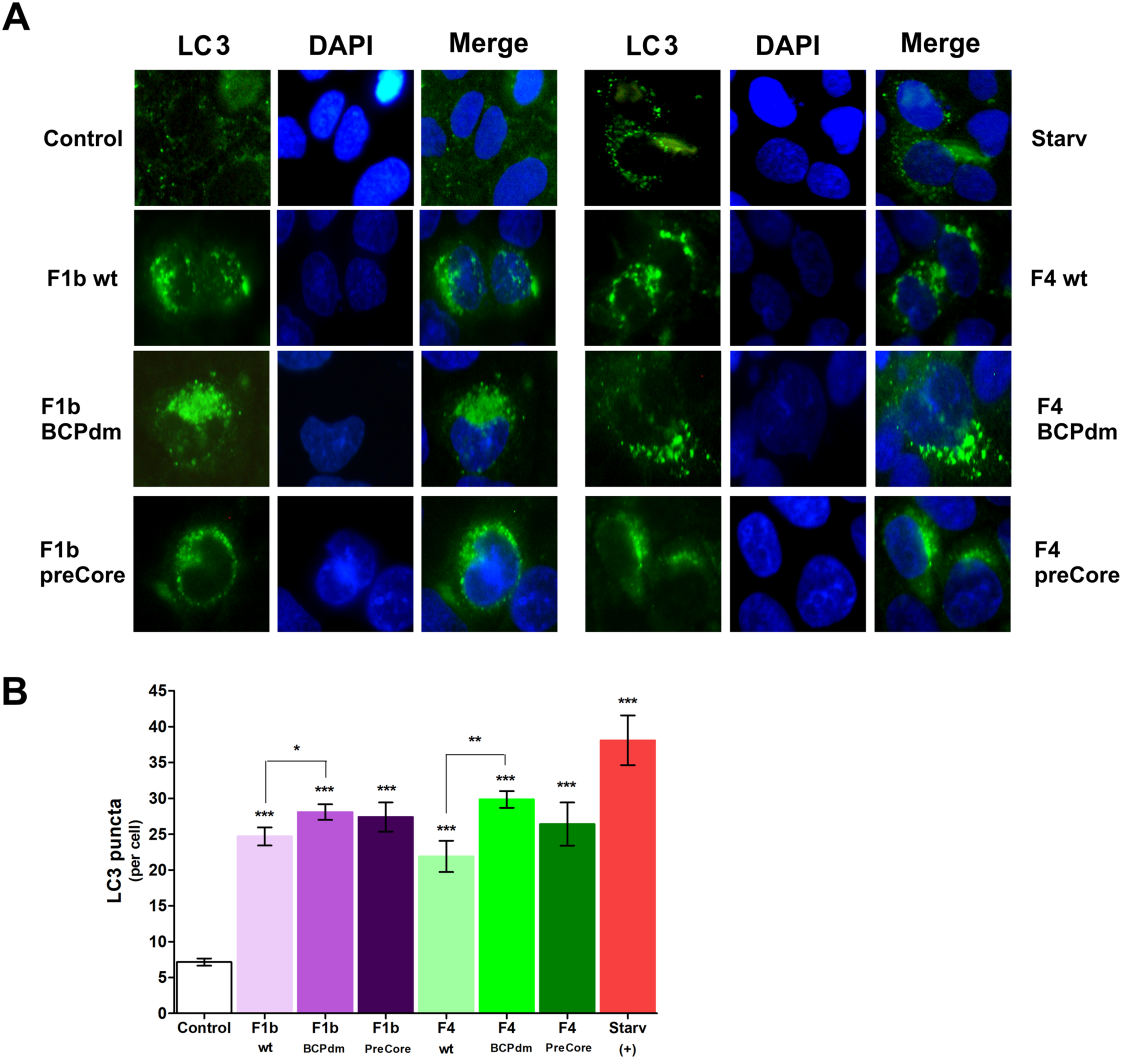
Detection of LC3 positive puncta in cells transfected with full-length HBV genomes. Huh-7 cells were either starved or transfected with pUC19 empty vector (control), F1b wt, F1b BCPdm, F1b preCore, F4 wt, F4 BCPdm and F4 preCore variants. Forty-eight hours post-transfection, cells were fixed and stained with anti-LC3 antibody. Nuclei were stained with DAPI, and distribution of LC3 protein was visualized with a fluorescence microscope. (A) Representative images are shown. (B) Quantification of LC3 positive puncta per cell. Shown values represent the mean ± standard deviation of three independent experiments. Statistically significant changes compared to control cells are indicated with asterisks above bars and statistically significant changes between the viral variants are indicated with asterisks above brackets. * *p* < 0.05; ** *p* < 0.005 and *** *p* < 0.0001.

A hallmark for the detection of autophagosomes is the analysis of LC3 protein. In absence of autophagy LC3 is diffusely localized in the cytosol, however, it is localized to autophagosomes during autophagy. HBV replication of sgts F1b and F4 significantly increased intracellular autophagosomes as demonstrated by accumulation of LC3-positive spot-like vesicles in the cell cytoplasm, in comparison to control cells. In addition, BCPdm replication induced significantly higher number of LC3-positive vesicles when compared to the wt viruses, for both sgts tested (Fig 2).

**Fig 3:**
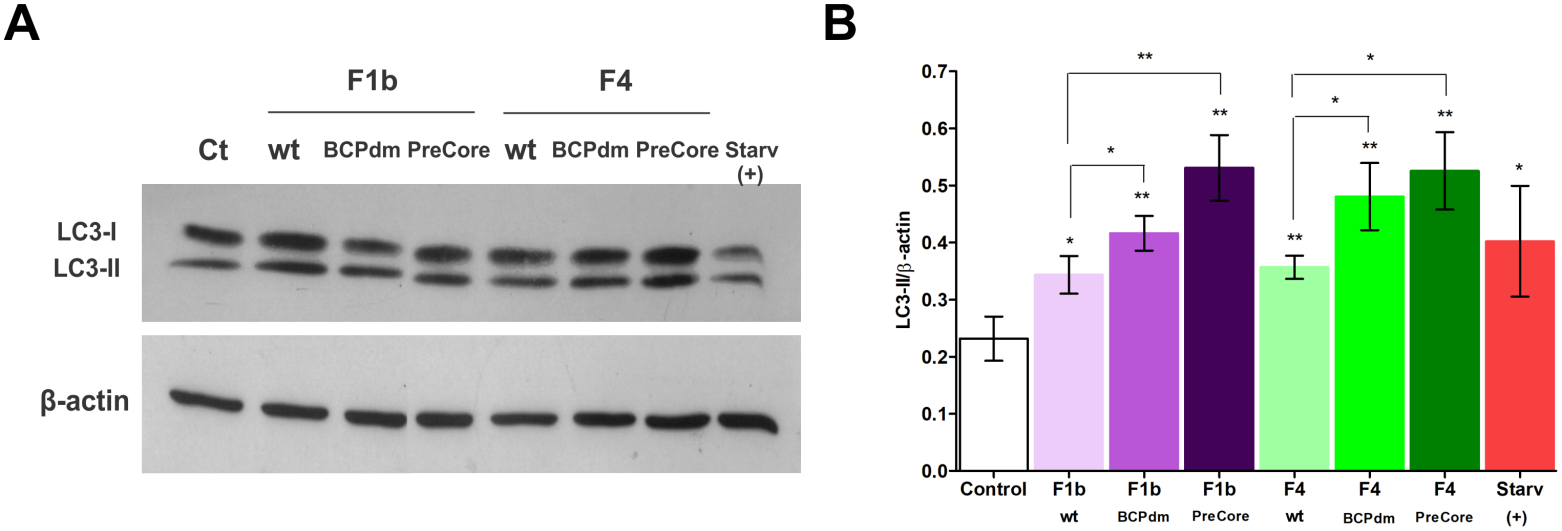
Conversion of LC3 in cells transfected with full-length HBV genomes. (A) Huh-7 cells were either starved or transfected with pUC19 empty vector (control), F1b wt, F1b BCPdm, F1b preCore, F4 wt, F4 BCPdm and F4 preCore variants. Forty-eight hours post-transfection, total cell proteins were extracted and expression levels of LC3-II were determined by Western Blot. (B) Relative intensity of the bands was quantified by normalization to β-actin, using ImageJ software. Shown values represent the mean ± standard deviation of three independent experiments. Statistically significant changes compared to control cells are indicated with asterisks above bars and statistically significant changes between the viral variants are indicated with asterisks above brackets. * *p* < 0.05; ** *p* < 0.005 and *** *p* < 0.0001.

To further confirm that HBV replication of sgts F1b and F4 induce autophagosome formation, the conversion of LC3 from the cytosolic form (LC3-I) to the lipidated autophagosome-associated form (LC3-II), was analyzed by Western Blot. HBV replication of sgts F1b and F4 significantly increased LC3-II levels in relation to control transfected cells, similar to that in starved Huh-7 cells. Additionally, an increased level of LC3-II was observed with BCPdm and preCore variants, compared to the wt, for both sgts tested (Fig 3).

It must be noted that no significant differences were observed in the accumulation of autophagosomes between sgts F1b and F4 for each analyzed variant.

Overall, these results suggest that HBV replication of sgts F1b and F4 induce accumulation of autophagosomes in Huh-7 cells. Furthermore, BCPdm as well as preCore mutants increased the accumulation of autophagosomes in relation to the wt virus.

### HBV replication of sgts F1b and F4 inhibits autophagic flux

**Fig 4:**
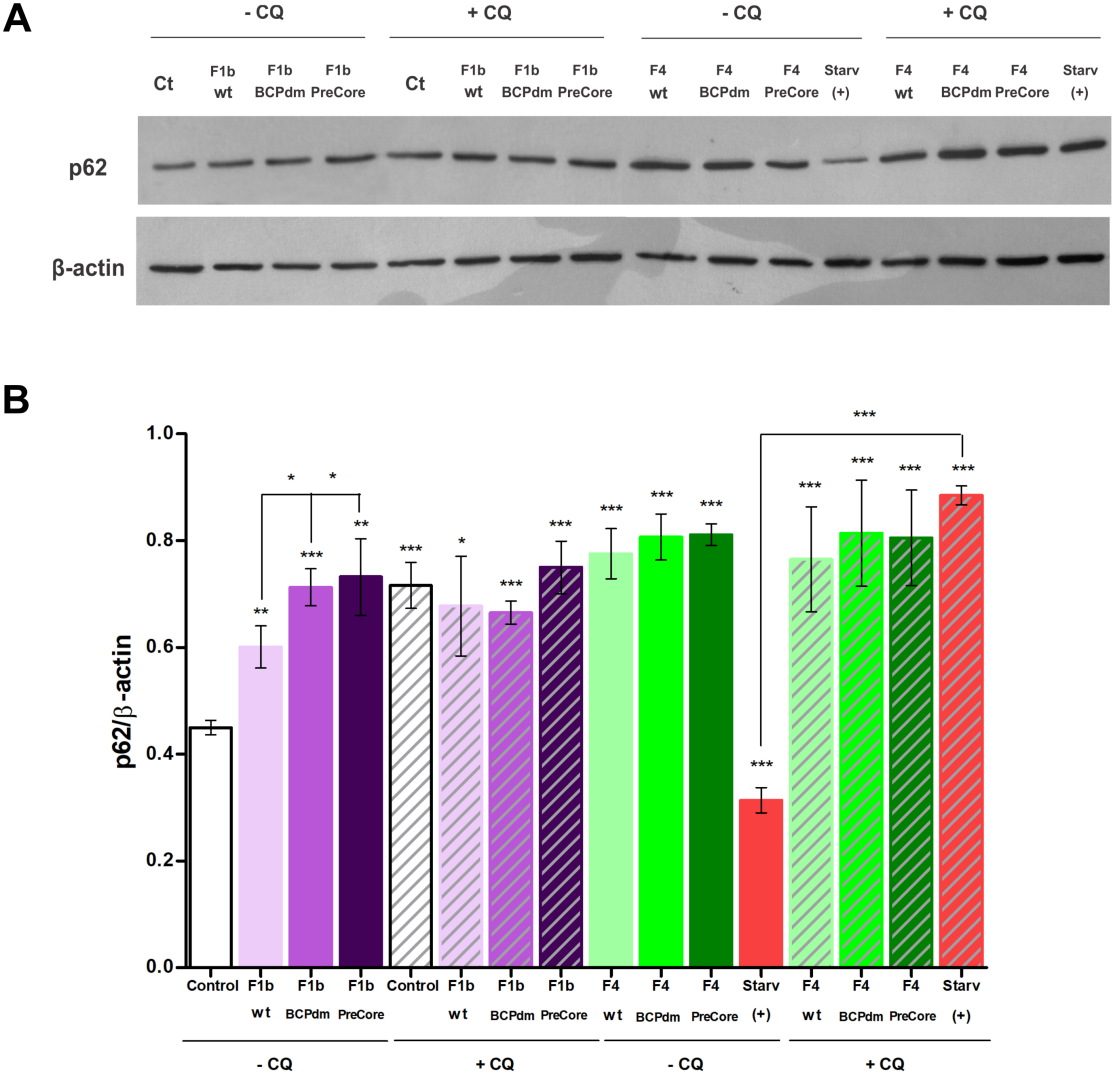
Inhibition of autophagic flux in cells transfected with full-length HBV genomes. (A) Huh-7 cells were either starved or transfected with pUC19 empty vector (control), F1b wt, F1b BCPdm, F1b preCore, F4 wt, F4 BCPdm and F4 preCore variants. Forty-eight hours post-transfection, cells were treated with or without CQ (100 μM) for 2 hours, total cell proteins were extracted and cellular levels of p62 were assessed by Western Blot. (B) Relative intensity of the bands was quantified by normalization to β-actin, using ImageJ software. Shown values represent the mean ± standard deviation of three independent experiments. Statistically significant changes compared to control cells are indicated with asterisks above bars and statistically significant changes between the viral variants and starved cells are indicated with asterisks above brackets. * *p* < 0.05; ** *p* < 0.005 and *** *p* < 0.0001.

In order to investigate whether HBV replication of sgts F1b and F4 induce a complete autophagic process (i.e., autophagic flux), protein levels of the autophagic substrate p62, a protein specifically degraded by the autophagy pathway, were analyzed by Western Blot. The results showed that HBV replication of sgts F1b and F4 increased the levels of p62, in contrast to starved Huh-7 cells where p62 levels were dramatically diminished. In addition, p62 level was significantly higher in sgt F1b BCPdm and preCore variants compared to sgt F1b wt, whereas no differences were observed among sgt F4 variants (Fig 4).

This phenomenon was further confirmed analyzing the levels of p62 in the presence of chloroquine, a lysosomotropic agent that prevents the acidification of lysosomes and blocks the downstream steps of autophagy [28]. If autophagy is induced, co-treatment with CQ will increase the p62 levels. On the contrary, if autophagy is blocked at downstream steps, p62 level will not be affected in the presence of CQ. As expected, we observed that CQ treatment had no effects on p62 levels in HBV sgts F1b and F4 transfected cells. By contrast, in starved cells, co-treatment with CQ increased p62 levels (Fig 4).

Taken together, these results suggest that autophagic flux is impaired in HBV sgts F1b and F4 transfected cells.

### HBV replication of sgts F1b and F4 impairs lysosomal acidification

**Fig 5:**
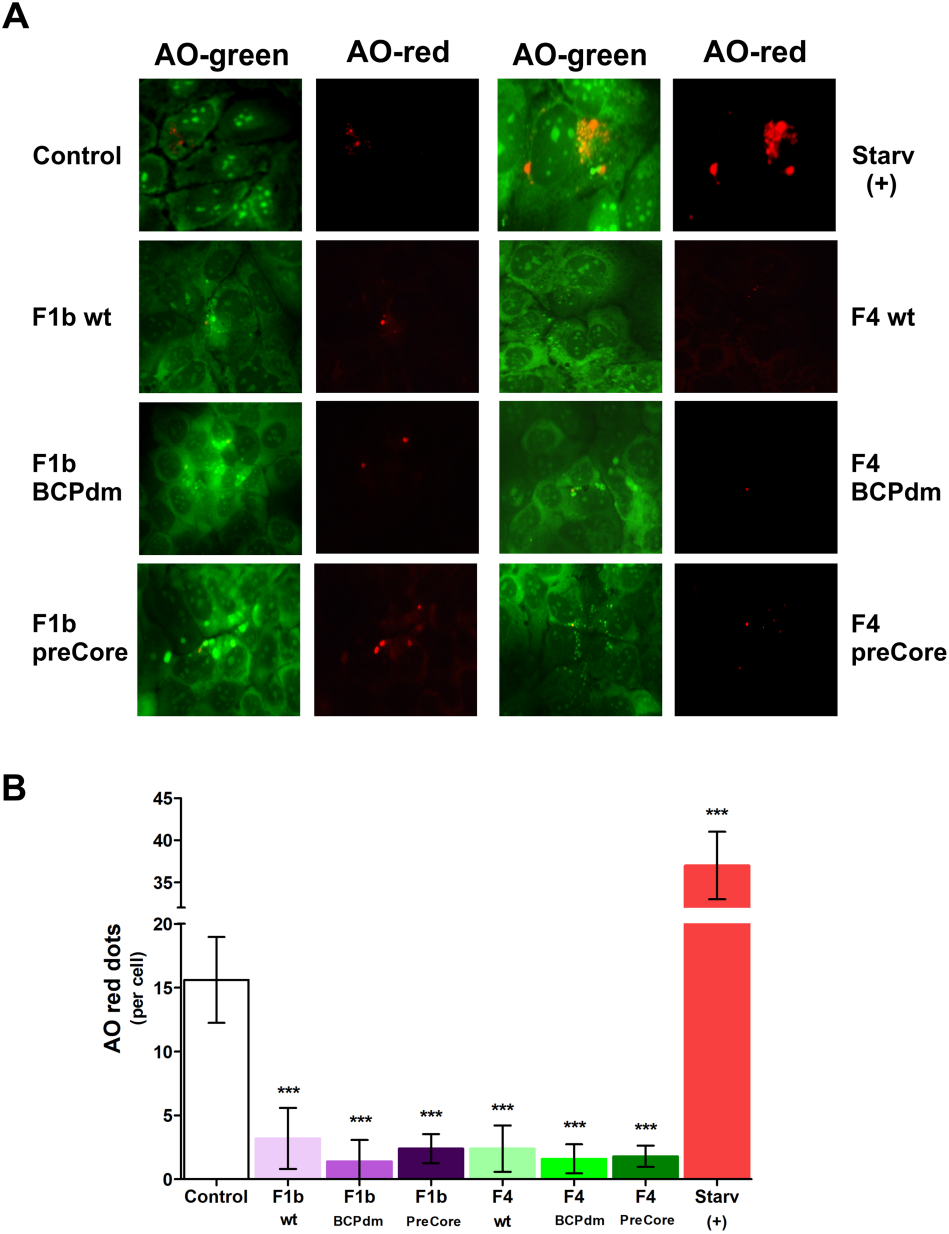
Detection of acidic vacuoles in cells transfected with full-length HBV genomes. Huh-7 cells were either starved or transfected with pUC19 empty vector (control), F1b wt, F1b BCPdm, F1b preCore, F4 wt, F4 BCPdm and F4 preCore variants. Forty-eight hours post-transfection, cells were fixed and stained with acridine orange (AO). AO was imaged through a 515/530-nm band-pass filter (green) or a 580-nm long-pass filter (red). (A) Representative images are shown. (B) Quantification of AO-red dots per cell. Shown values represent the mean ± standard deviation of three independent experiments. Statistically significant changes compared to control cells are indicated with asterisks above bars. *** *p* < 0.0001.

Since acidification is required for the maturation and activation of lysosomal enzymes, the maintenance of acidity is a hallmark of functionality in mature lysosomes and autolysosomes. To determine whether HBV inhibition of autophagic protein degradation was due to an impairment of lysosomal acidification, the presence of acidic vacuoles (lysosomes and autolysosomes) was studied by assessing their retention of AO. The non-protonated monomeric form of AO emits green fluorescence in the cytosol. But, when the dye enters acidic lysosomes, the protonated form becomes trapped in aggregates that fluoresce bright red. HBV replication of sgts F1b and F4 dramatically decreased the number of AO red dots per cell (Fig 5), indicating a reduced number of acidified compartments. In contrast, a significant increase in the number of AO red dots per cell was observed in starved Huh-7 cells.

**Fig 6:**
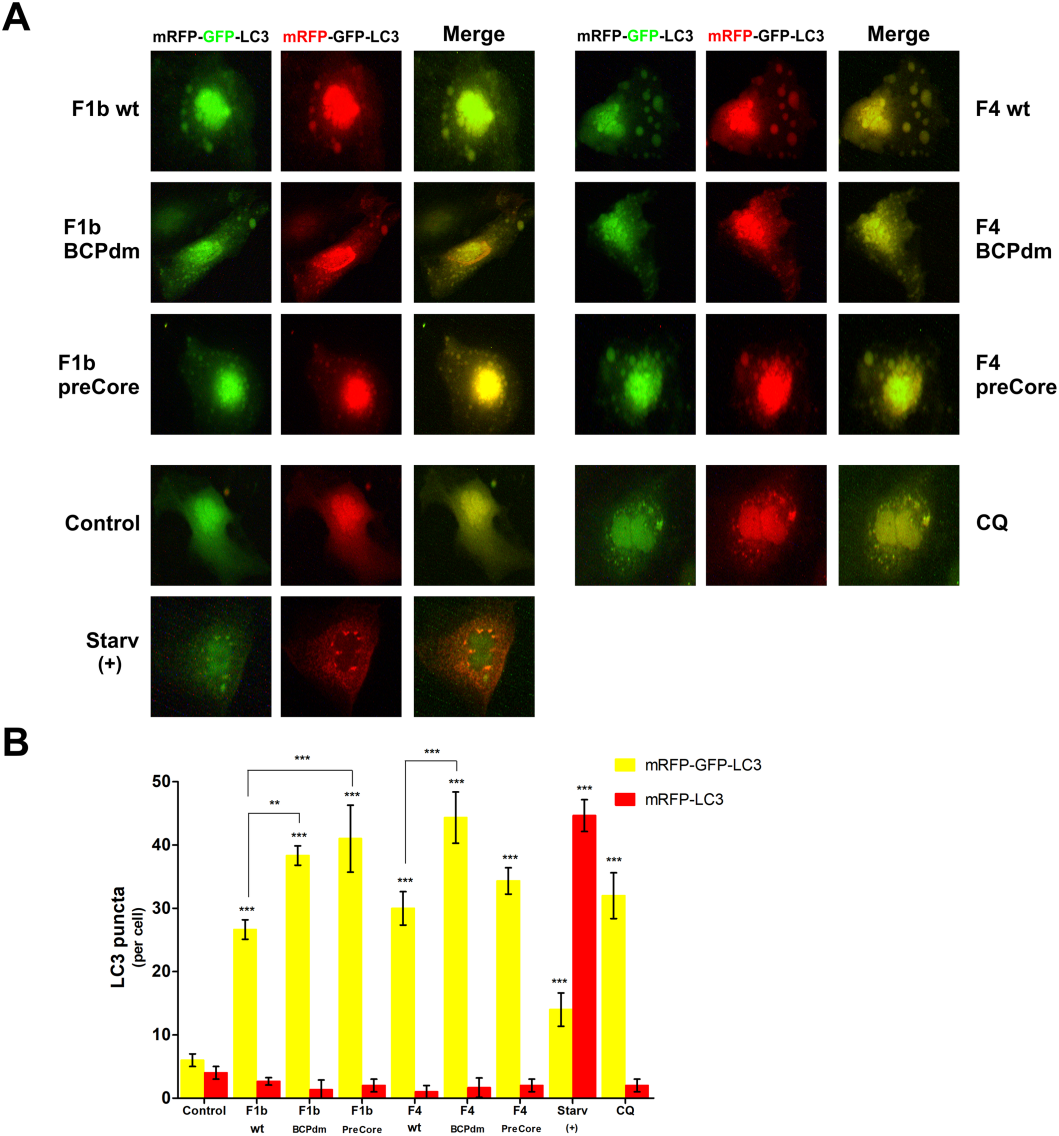
Expression of the tandem protein mRFP-GFP-LC3 in cells transfected with full-length HBV genomes. Huh-7 cells transfected with pUC19 empty vector (control), F1b wt, F1b BCPdm, F1b preCore, F4 wt, F4 BCPdm and F4 preCore variants, starved or treated with CQ (100 μM) were transiently transfected with mRFP-GFP-LC3 expression plasmid. Forty-eight hours post-transfection, cells were fixed and imaged through a 515/530-nm band-pass filter (green) or a 580-nm long-pass filter (red). (A) Representative images are shown. (B) Quantification of yellow puncta (mRFP-GFP-LC3 positive) and red puncta (mRFP-LC3 positive) per cell. Shown values represent the mean ± standard deviation of three independent experiments. Statistically significant changes compared to control cells are indicated with asterisks above bars and statistically significant changes between the viral variants are indicated with asterisks above brackets. ** *p* < 0.005 and *** *p* < 0.0001.

Lysosomal acidification was further examined by expression of the tandem protein mRFP-GFP-LC3. Under non-lysosomal and neutral pH condition, both mRFP and GFP fluoresce. However, the low pH in the lumen of the lysosome quenches the GFP signal, but not the mRFP signal. Therefore, the red puncta that overlay with the green puncta (merged as yellow) are indicators of autophagosomes, whereas the solely red puncta are indicative of autolysosomes. We found that in starved cells, most of the puncta lost the GFP signal and retained the mRFP signal. But in cells transfected with HBV sgt F1b and F4 genomes, only yellow punctuate fluorescence was markedly increased, similar to that seen following CQ treatment, indicating a reduced number of autolysosomes. In addition, a significant increase in the number of yellow puncta was observed with BCPdm and sgt F1b preCore variants, compared to the wt virus (Fig 6).

Together, these results suggest that HBV replication of sgts F1b and F4 impairs lysosomal acidification in Huh-7 cells.

## Discussion

In this study, we report evidence of an incomplete autophagy process induced by HBV sgts F1b and F4 replication in Huh-7 cells. These sgts, native from Latin America, are responsible for most of the new acute and chronic infections in our country [8,10], and have been scarcely studied so far.

Autophagy is a protective process that recycles damaged organelles and unwanted proteins to ensure cell viability under starvation and stress conditions. Moreover, autophagy has also been regarded as a key component of the innate immune system. Induction of autophagy results in the elimination of invading pathogens, however, some microbes, including viruses, have evolved strategies to manipulate autophagy for their own benefit [22]. It has been proposed that autophagy could contribute to viral infections by numerous mechanisms. For example, autophagosomes could serve as scaffolds for viral replication and assembly. Moreover, persistent viral replication would benefit from autophagy process, since it contributes to cell survival. Finally, viruses have evolved to regulate autophagy so as to interrupt autophagosome maturation and thus escape from lysosomal degradation [21,29].

In the present work, we demonstrate that HBV replication of sgts F1b and F4 induce accumulation of autophagosomes in Huh-7 cells. The number of autophagosomes in the cytosol can be increased by two mechanisms: increased autophagosome synthesis by upstream processes (and hence an increase in autophagic flux) or blockage of lysosomal degradation at a later stage (and hence a decrease in autophagic flux). In our study, no autophagic protein degradation was observed in HBV transfected cells, mimicking the action of CQ, which indicates that HBV replication would inhibit autophagic flux at later stages of the autophagic process.

This result is in agreement with previous studies reporting that HBV replication induced an incomplete autophagy pathway in hepatic and hepatoma cells [30–32]. Therefore, our results confirm previous *in vitro* and *in vivo* studies, and show that genotype F, one of the less characterized HBV genotypes, behaves as the other genotypes in relation to its capacity to regulate autophagy.

In this study, no significant differences were observed in the modulation of the autophagy process between sgts F1b and F4, two subgenotypes known to possess different biological characteristics. This might indicate that HBV-mediated regulation of autophagy in human hepatocytes is independent of the viral subgenotype.

In addition, our results also suggest that the inhibition of autophagy flux in Huh-7 cells might be due to an impairment of lysosomal acidification. This result is consistent with the previous finding of Liu et al. [30], in which they demonstrated that HBV, and HBV X protein in particular, impaired lysosomal degradative capacity by disturbing its acidification without influencing the fusion of autophagosomes and lysosomes.

Therefore, using this strategy, HBV would have a dual effect on the regulation of the autophagic pathway. On one hand, HBV could induce autophagosome accumulation to increase viral DNA replication. In this respect, several studies have indicated that HBV induction of autophagosomes contributes to virus production, and inhibition of autophagosome formation suppresses HBV replication [23,33]. Additionally, the increase in the number of autophagosomes might play a key role in the acquisition of the viral envelope, supporting the idea that autophagosome-derived membranes could be used as a source of membranes for viral envelopment [25]. On the other hand, HBV might also inhibit later stages of autophagy, suppressing lysosomal activity, as a survival mechanism to escape direct destruction and thus avoid the generation of antigenic peptides and/or attenuate antigen presentation.

Thus, HBV modulation of autophagy in hepatic cells of chronically infected patients may play an important role in HBV pathogenesis, increasing virus production and maintaining the viability of infected cells. In addition, increasing evidence suggest that defects in autophagy might be associated with the development of cancer. It has been shown that accumulation of p62, either by the loss of essential autophagy genes or inhibition in lysosomal function, may contribute to the development of HCC [34]. It was demonstrated that p62 excess leads to the formation of aggregates that cause DNA damage and stimulate a pro-tumorigenic inflammatory response [35–37]. Therefore, the activation of autophagy to increase viral replication, as well as the inhibition of lysosomal activity would play a role in HBV pathogenesis, contributing to the development of liver disease and HCC.

Several studies have extensively described the occurrence of mutations in BCP and preCore regions in the late stage of HBV chronic infection, as well as their implication in the regulation of viral replication and protein expression [38–41]. However, there is a paucity of information regarding the underlying mechanisms that associate these mutations with the progression of chronic infection.

In this study, BCP as well as preCore mutants significantly increased the accumulation of autophagosomes and p62 in comparison to the wt genomes, which suggests an autophagy blockage enhancement caused by these variants. This is the first report analyzing the effect that BCP and preCore mutations exert on this process. The results obtained in this work suggest that throughout adaptation of the virus to host environment, selection of these mutations could be evolutionarily driven to support viral persistence and collaterally contribute to the pathogenesis of infection.

Increasing evidences indicate that autophagy plays a crucial role in the regulation of apoptosis [42]. Generally, autophagy reduces the propensity of cells to undergo apoptosis, degrading and recycling damaged organelles, or protein aggregates through lysosomal degradation [43]. However, when there is a blockage in the autophagy process (e.g. impairment of lysosomal acidification), apoptosis is then activated. In a previous study we have demonstrated that HBV sgts F1b and F4 replication induced cell death by apoptosis in Huh-7 cells. Moreover, BCPdm and preCore mutants induced higher levels of apoptosis than wt virus [44]. It might be possible that the autophagy blockage induced by HBV sgts F1b and F4 replication, associated with the increase of intravesicular pH, finally conclude in the induction of apoptosis of transfected cells; and the higher levels of apoptosis induced by BCPdm and preCore mutants could be associated with the autophagy blockage enhancement caused by these variants.

Overall in the present study we have demonstrated that HBV sgts F1b and F4 replication induced accumulation of autophagosomes without autophagic protein degradation in Huh-7 cells, indicating a blockage of autophagic flux at later stages of the process. This inhibition of autophagy flux might be due to an impairment of lysosomal acidification in Huh-7 cells, which finally conclude in the induction of apoptosis of transfected cells. Moreover, HBV-mediated autophagy modulation was independent of the viral sgt and enhanced in viruses with BCP and preCore naturally occurring mutations.

The results of this work help to understand the molecular mechanisms by which different HBV variants contribute to the pathogenesis of chronic HBV infections. This study is the first to describe the role that two highly prevalent naturally occurring mutations exert on the modulation of HBV-induced autophagy. Furthermore, this work provides new pieces of knowledge that contribute to the biological and molecular characterization of genotype F, native of Latin American population, and responsible for most HBV chronic infections in Argentina.

## Supporting information

S1 Fig Alignment of nucleotide sequences corresponding to the basal core promoter region (1742 to 1849, black rectangle) and preCore region (1814 to 1901, blue rectangle) of F1b wt, F1b BCPdm, F1b preCore, F4 wt, F4 BCPdm and F4 preCore variants. The nucleotide variation at 1762, 1764 and 1896 positions are indicated by open boxes.
(TIF)
