# Supplementary figures and images for: HBV subgenotypes F1b and F4 replication induces an incomplete autophagic process in hepatocytes: Role of BCP and preCore mutations
Source: PLoS One. 2018 May 8;13(5):e0197109. doi: 10.1371/journal.pone.0197109 (PMC5940199; doi:10.1371/journal.pone.0197109)

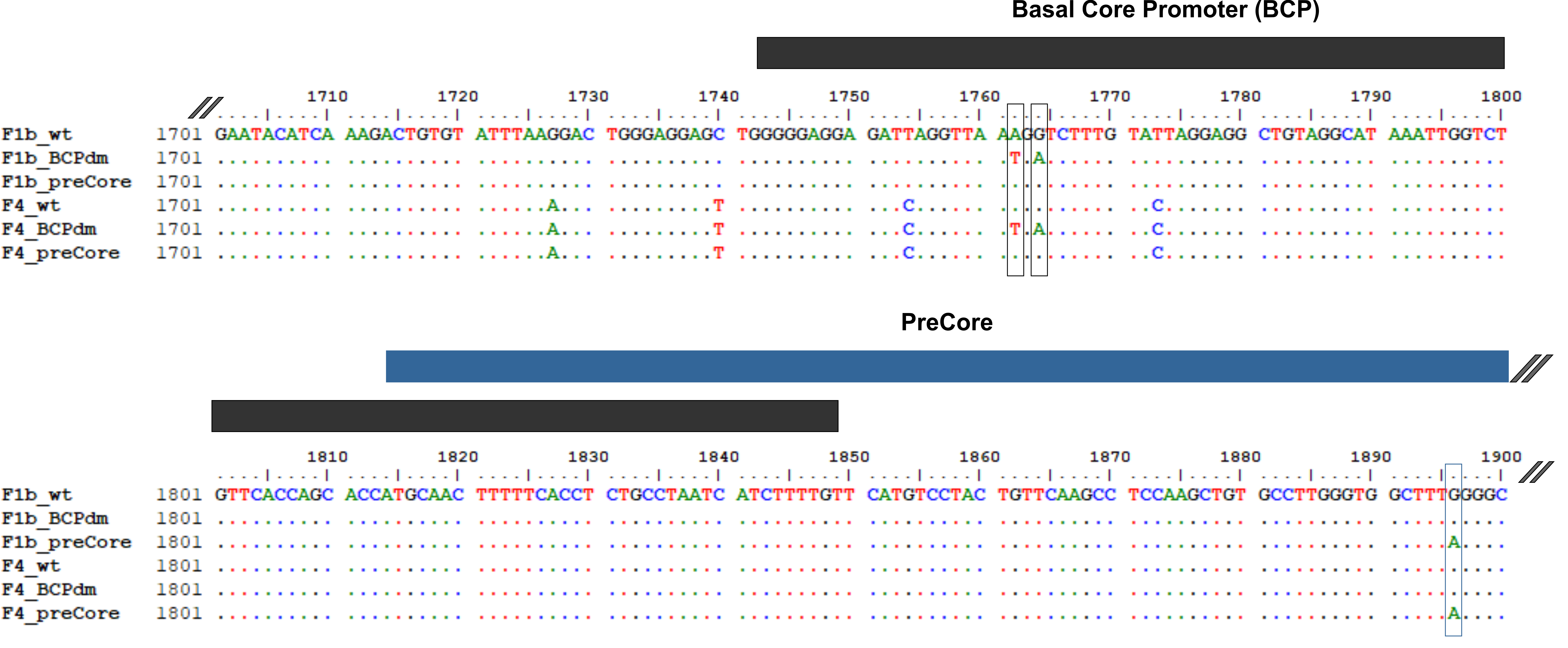

Supplement: S1 Fig — The nucleotide variation at 1762, 1764 and 1896 positions are indicated by open boxes. (TIF) [file pone.0197109.s001.tif]
